# Supplementary material for: Ultrasensitive Alzheimer’s disease biomarker detection with nanopillar photonic crystal biosensors
Source: Optica. 2025 Oct 2;12(10):1587–96. doi: 10.1364/OPTICA.566672 (PMC12510715; doi:10.1364/OPTICA.566672)
Supplement: Supplementary file 1 [file optica-12-10-1587-s001.pdf]

Supplemental Document

OPTICA

Ultrasensitive Alzheimer's disease biomarker detection with nanopillar photonic crystal biosensors: supplement

Guilherme S. Arruda,[1,*] Katie Morris,[2] Augusto Martins,[2] Yue Wang,[2] Sian Sloan-Dennison,[3] Duncan Graham,[3] Steven D. Quinn,[2,4] Emiliano R. Martins,[1] and Thomas F. Krauss[2,4]

[1] *São Carlos School of Engineering, Department of Electrical and Computer Engineering, University of São Paulo, São Carlos, SP 13566-590, Brazil*
[2] *School of Physics, Engineering and Technology, University of York, York YO10 5DD, UK*
[3] *Department of Pure and Applied Chemistry, University of Strathclyde, Technology and Innovation Centre, Glasgow G1 1RD, UK*
[4] *York Biomedical Research Institute, University of York, York YO10 5DD, UK*
[*] *guilherme.simoneti.arruda@usp.br*

This supplement published with Optica Publishing Group on 2 October 2025 by The Authors under the terms of the Creative Commons Attribution 4.0 License in the format provided by the authors and unedited. Further distribution of this work must maintain attribution to the author(s) and the published article's title, journal citation, and DOI.

Supplement DOI: https://doi.org/10.6084/m9.figshare.29979910

Parent Article DOI: https://doi.org/10.1364/OPTICA.566672

Supplementary Information: Ultrasensitive Alzheimer Biomarker Detection with Nanopillar Photonic Crystal Biosensors

Guilherme S. Arruda[1], Katie Morris[2], Augusto Martins[2], Yue Wang[2], Sian Sloan-Denisson[3], Duncan Graham[3], Steven D. Quinn[2,4], Emiliano R. Martins[1], Thomas F. Krauss[2,4].

Filiation:
1 São Carlos School of Engineering, Department of Electrical and Computer Engineering, University of São Paulo, São Carlos – SP, 13566-590, Brazil
2 School of Physics, Engineering and Technology, University of York, York, YO10 5DD, United Kingdom.
3 Department of Pure and Applied Chemistry, University of Strathclyde, Technology and Innovation Centre, Glasgow G1 1RD, United Kingdom.
4 York Biomedical Research Institute, University of York, York, YO10 5DD, United Kingdom.

Keywords: Alzheimer's Disease, nanoparticles, nanopillars, biosensors, photonic crystals, high-Q, low-cost.

1. Physics of the dimer configuration.

The dielectric nano-pillar dimer circumvents the trade-off between Q-factor and sensitivity, offering the ability to tune the Q-factor in a wide range. To understand the physical origin of this feature, consider the Fourier Series expansion, of the permittivity distribution $\varepsilon(\boldsymbol{r})$ of a photonic crystal:

$$\varepsilon(\boldsymbol{r}) = \sum_{qp} \epsilon[q,p] e^{-j\boldsymbol{G_{qp}}\cdot\boldsymbol{r}} \quad \text{(S1)}$$

where $\boldsymbol{G_{qp}}$ is the reciprocal lattice vector, $\boldsymbol{r}$ is the in-plane position vector, $\epsilon[q,p]$ represents the Fourier components of order q and p. The coupling between radiating waves and the waveguided Bloch modes is mediated by the structure's first-order Fourier components [1,2], that is, by $\epsilon[1,0]$ and $\epsilon[0,1]$. Since the Q-factor depends on this coupling, it can be controlled by tuning $\epsilon[1,0]$ and $\epsilon[0,1]$. This connection between the Fourier components $\epsilon[q,p]$ and the Q-factor can be further clarified by considering a photonic crystal (PhC) slab consisting of a square array of holes (lattice period a_h, holes' diameter W_h) patterned into a dielectric film on top of glass (Fig. S1a and inset of Fig. S1b). For illustration, we consider an ideal non-absorbing aSi ($n = 3.5$) film layer. For a square unit cell (inset of Fig. S1b), the first order Fourier component $\epsilon_h[1] \equiv \epsilon_h[1,0] = \epsilon_h[0,1]$ is given by [3]:

$$\epsilon_h[1] = \frac{2(\varepsilon_h - \varepsilon_{aSi})(1-FF)J_1\left(\frac{\pi W_h}{a_h}\right)}{\frac{\pi W_h}{a_h}} \quad \text{(S2)}$$

where ε_h and ε_{aSi} are the dielectric constants of the material inside the hole (typically water for sensing applications) and the aSi film respectively; FF is the Fill Factor representing the ratio of the area filled by the high index material (the slab) to the area filled by the low index material (the holes) and J_1 is the first order Bessel function of the first kind.

According to equation S2, once the materials have been chosen, the only degree of freedom to control the $\epsilon_h[1]$ component (and consequently the Q-factor) is FF. An example of the relationship between Q-factor and FF is shown in Fig. S1b. Note that the Q-factor goes to

infinity as FF approaches unity ($\epsilon_h[1]\rightarrow 0$), which corresponds to a slab without holes. In this case, the waveguide mode no longer couples to radiation modes, and hence the Q-factor is infinite. In general, the Q-factor scales with the inverse of $\epsilon_h[1]$ (black solid line in Fig. S1c), since a reduction of the latter implies reduced energy leakage of the mode into radiating waves. Finally, the higher the FF, the smaller the nanohole and, consequently, the more confined the mode within the slab, resulting in higher effective index (n_{eff}) and lower sensitivity to the analyte on its surface. This trend can be seen in Fig. S1b-c, which shows an increase of n_{eff} with FF. Thus, there is a clear trade-off between Q-factor and sensitivity in the design of nanoholes array sensors.

This trade-off can be circumvented using dimer nano-pillars. To clarify the role of the dimers, first consider an array of single nano-pillars (period $\mathrm{a_p}$, diameter W_p, see Fig. S1d and inset of Fig. S1e). Its first order Fourier component ($\epsilon_p[1] = \epsilon_p[1,0] = \epsilon_p[0,1]$) is given by:

$$\epsilon_p[1] = \frac{2(\varepsilon_{aSi}-\varepsilon_h)FFJ_1\left(\frac{\pi W_P}{\mathrm{a}_P}\right)}{\frac{\pi W_P}{\mathrm{a}_P}} \tag{S3}$$

Once again, the only degree of freedom to control $\epsilon_p[1]$ is the FF. Contrary to what has been observed in the hole array (Fig S1a), the Q-factor in the pillar array only shows a modest dependence on FF (Fig. S1e). Importantly, the FF is bound between ~0.17 and ~0.8. The upper bound comes from geometrical limitations (the pillars touch each other), while the lower bound arises from waveguiding limitations (below ~0.17 the n_{eff} is too low to support a guided mode, see Fig. S1f). Consequently, the Q-factor of pillar arrays are typically orders of magnitude lower than that of hole arrays (note the difference in the scales of Fig. S1b-e).

The dimer configuration (periods a_y and a_x, diameter W_d, Fig. S1g-h) solves the trade-off problem by introducing an additional geometrical degree of freedom to independently control the first Fourier component of the array permittivity distribution. This control is achieved by tuning the centre-to-centre distance of the pillars g_c, which relates to the first Fourier component of the dimer permittivity distribution, $\epsilon_d[0,1]$, as (see section 2 for a deduction):

$$\epsilon_d[0,1] = 2(\varepsilon_{aSi} - \varepsilon_h)FF\frac{J_1\left(\frac{\pi W}{\mathrm{a}_y}\right)}{\frac{\pi W}{\mathrm{a}_y}}\cos\left(\frac{\pi g_c}{\mathrm{a}_y}\right) \tag{S4}$$

As shown in Fig. S1h, the Q-factor of the mode mediated by $\epsilon_d[0,1]$ (equation S4) increases monotonically following the nanopillar centre-to-centre distance g_c. This increase is directly linked to the reduction in the coupling component $\epsilon_d[0,1]$ for increasing g_c, as shown in Fig. S1i. Explicitly, the smaller $\epsilon_d[0,1]$ (for wider post separation), the higher the Q-factor. As the separation approaches $\mathrm{a}_y/2$, the Q-factor of the structure diverges in the limit that $g_c = \mathrm{a}_y/2$. At this condition, the period of the structure is halved and the mode no longer couples to radiation because $\epsilon_d[0,1]$ vanishes, which effectively closes the cavity (Q-factor diverges to infinity).

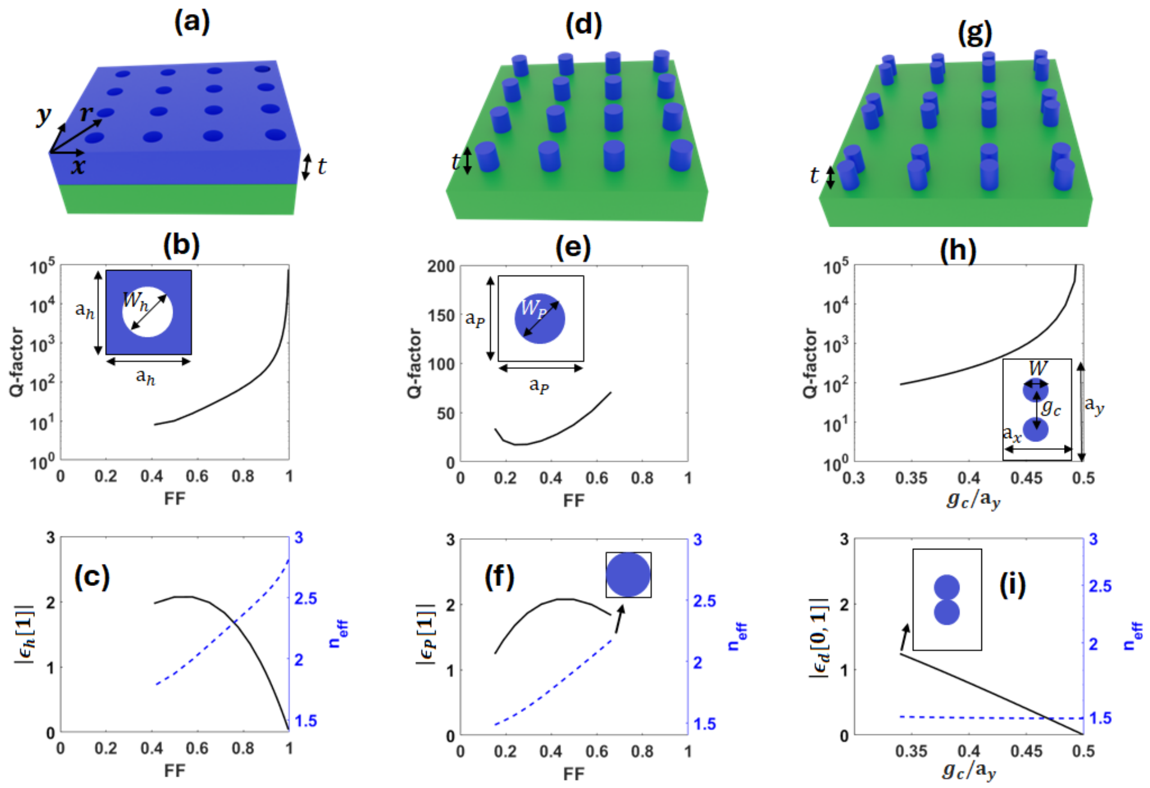

Figure S1: Schematic of the (a) nanoholes patterned in an aSi ($n =$ 3.5) film, (d) single and (g) dimer aSi nanopillars. The thickness of the aSi film and pillars is $t = 100$ nm. All structures are assumed to sit on top of a glass substrate ($n =$ 1.45, green) and immersed in water ($n =$ 1.33, white background). (b, e) Relationship between Q-factor and the fill factor of holes and pillars FF. (h) Relationship between the Q-factor and the dimer pillar centre-to-centre gap distance g_c. (Insets b, e and h) Unit cells of the nanoholes, single and dimer pillar structures with their corresponding geometric parameters. (c, f) Nano-holes and single pillar first Fourier components $\epsilon_h[1]$ and $\epsilon_P[1]$ (black solid lines), respectively, and their mode effective index n_{eff} (blue dashed lines) dependence on their unit cell FF. The maximum FF of the pillars is limited when the pillar's diameter is equal to its period, near $FF = 0.79$, as indicated in the inset of (f), while the minimum FF is limited by the waveguiding condition (the mode's n_{eff} approaches it cut-off value near 1.5). (i) Dimer pillar first Fourier component $\epsilon_d[0,1]$ (black solid line) and its associated mode n_{eff} (blue dashed line) dependence on g_c. The minimum distance is limited by the two pillars touching one another each other, around $g_c = 0.34\mathrm{a}_y$, as indicated in the inset. The nano-holes and single pillar array periods ($\mathrm{a}_h$and a_p) were adjusted for the structure to support a mode at $\lambda_0 = 750$ nm. For the dimer structure, $\mathrm{a}_y = 500$ nm, $\mathrm{a}_x = 320$ nm and $W = 170$ nm.

2. Derivation of equation 1 of the main text.

First consider the unit cell of a rectangular lattice containing a circle at its centre with periods a_x and a_y in the corresponding $\boldsymbol{x}$ and $\boldsymbol{y}$ directions, respectively, as illustrated in Fig. S2a, with Fourier series components $\epsilon_p[q,p]$, where q and p are the components' orders along the **x** and **y** directions, respectively. From the shifting property of the Fourier series, a displacement in real space (Fig. S2b) imposes a phase modulation on the Fourier components:

$$\epsilon'_p[q,p] = \epsilon_p[q,p]\left[e^{-i\left(q\frac{2\pi}{\mathrm{a}_x}x_1+p\frac{2\pi}{\mathrm{a}_y}y_1\right)}\right] \quad \text{(S5)}$$

where $\epsilon'_p[q,p]$ are the Fourier components of the displaced circle and $\boldsymbol{r_1} = x_1\boldsymbol{x} + y_1\boldsymbol{y}$ is its centre position. Following equation S5, the Fourier components of two displaced circles with the same diameter (Fig. S2c) is given by:

$$\epsilon_d[q,p] = \epsilon_p[q,p]\left[e^{-i\left(q\frac{2\pi}{a_x}x_1+p\frac{2\pi}{a_y}y_1\right)} + e^{-i\left(q\frac{2\pi}{a_x}x_2+p\frac{2\pi}{a_y}y_2\right)}\right] \tag{S6}$$

where $\boldsymbol{r_2} = x_2\boldsymbol{x} + y_2\boldsymbol{y}$ is the displacement vector of the second circle. Assuming that both circles are symmetrically displaced along the y axis, we obtain:

$$x_1 = x_2 = 0 \tag{S7}$$

$$y_1 = -y_2 = \frac{g_c}{2} \tag{S8}$$

where g_c is the center-to-center distance between the two circles. Using equations S3 and S4 in S6, it follows that the Fourier components of the dimer structure are given by

$$\epsilon_d[q,p] = \epsilon_p[q,p]\cos\left(p\frac{\pi}{a_y}g_c\right) \tag{S9}$$

The permittivity Fourier components of a periodic array of circles ($\epsilon_p[q,p]$) with diameter W, dielectric constant $\varepsilon = \varepsilon_a$ and immersed in a material with $\varepsilon = \varepsilon_b$ are given by [3]:

$$\epsilon_p[q,p] = \begin{cases} \dfrac{2(\varepsilon_a-\varepsilon_b)\Upsilon J_1\left(\frac{G_{qp}W}{2}\right)}{\frac{G_{qp}W}{2}} & (\boldsymbol{q},\boldsymbol{p}) \neq (\boldsymbol{0},\boldsymbol{0}) \\ \varepsilon_a\Upsilon + \varepsilon_b(1-\Upsilon) & (\boldsymbol{q},\boldsymbol{p}) = (\boldsymbol{0},\boldsymbol{0}) \end{cases} \tag{S10}$$

where:

$$G_{qp} = \sqrt{\left(q\frac{2\pi}{a_x}\right)^2 + \left(p\frac{2\pi}{a_x}\right)^2} \tag{S11}$$

is the magnitude of the reciprocal lattice vector $\boldsymbol{G_{qp}}$ for the [q,p] component, Υ is the circle area filling factor and J_1 is the first order Bessel function of the first kind. For the rectangular array of dimer aSi pillars of Fig. S1g-h, we have: $\varepsilon_a = \varepsilon_{aSi}$ (aSi pillars), $\varepsilon_b = \varepsilon_c$ (water medium), $\Upsilon = FF/2$, where FF is the dimer structure filling factor. From equations S9-S11, $\epsilon_d[0,1]$ and $\epsilon_d[1,0]$ are given by

$$\epsilon_d[0,1] = 2(\varepsilon_{aSi} - \varepsilon_c)FF\frac{J_1\left(\frac{\pi W}{a_y}\right)}{\frac{\pi W}{a_y}}\cos\left(\frac{\pi g_c}{a_y}\right) \tag{S12}$$

and

$$\epsilon_d[1,0] = 2(\varepsilon_{aSi} - \varepsilon_h)FF\frac{J_1\left(\frac{\pi W}{a_x}\right)}{\frac{\pi W}{a_x}} \tag{S13}$$

where equation S12 is equation 1 of the main text (and equation S4 of this Supplementary Information).

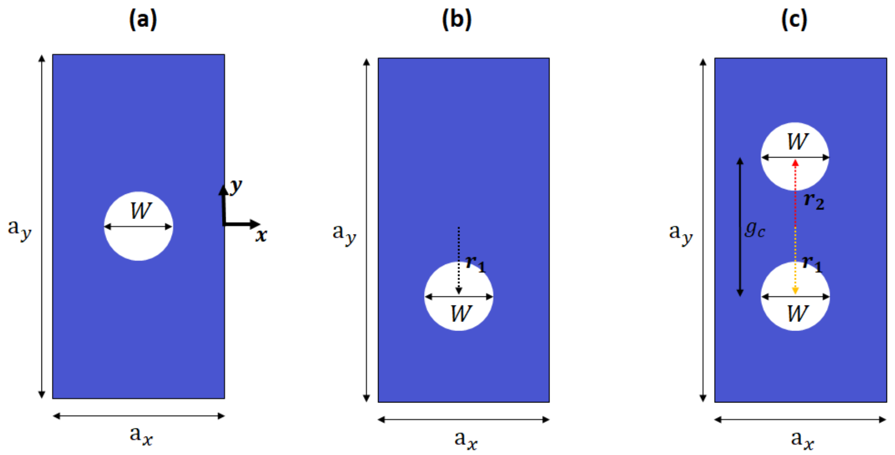

Figure S2: Schematic of the unit cell with a single circle on its centre (a), a displaced circle (b) and two symmetrically displaced circles (c).

3. Band diagrams of nano-holes, single and dimer pillars.

Fig. S3 shows the transmittance colour maps as a function of wavelength and in-plane wavevector ($\boldsymbol{k}$) along the $\Gamma \rightarrow M$ direction [4] for the structures shown in Fig. S1, namely, a square array of nano-holes etched into a thin aSi film (Fig. S3a), a square array of aSi nano-pillars (Fig. S3b) and the rectangular dimer array of aSi nano-pillars (Fig. S3c). The band diagrams can be readily seen on the transmittance colour maps as sharp Fano resonance lines (in blue, i.e, low transmittance values). The bands were calculated using an in-house implementation of the Rigorous Wave Coupled Analysis assuming incoming electric fields towards both $\boldsymbol{x}$ (E_x, Fig. S3d-f) and $\boldsymbol{y}$ (E_y, Fig. S3g-i) directions [3,5]. Note that all structures support leaky Bloch modes and genuine BICs (highlighted by the black dots), as evidenced by the vanishing bands at the Γ point (where the parallel component of the wavevector $k = 0$). The resonance used for the sensor is highlighted by the white arrow in Fig. S3f.

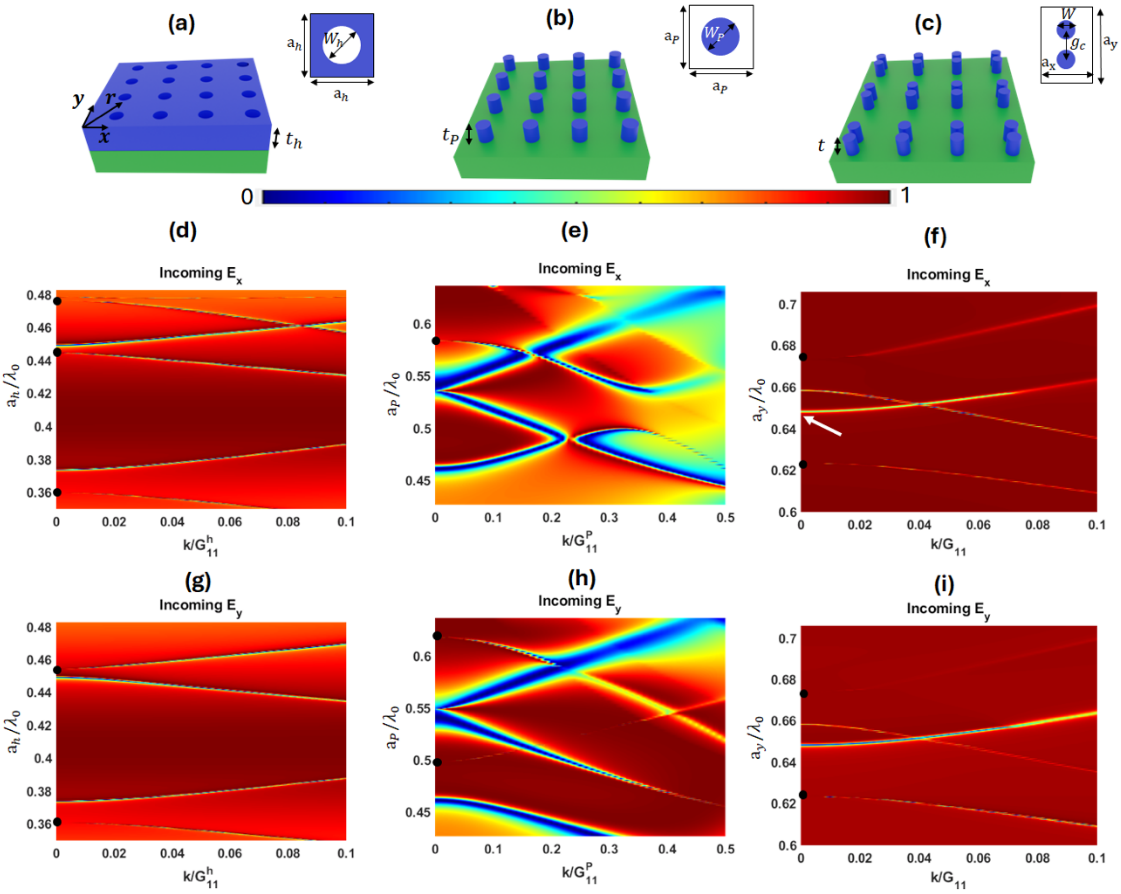

Figure S3: Schematic of the array of nano-holes patterned into a thin aSi film (a), aSi single pillar (b) and dimer pillars (c) with their respective unit cells and geometric parameters as insets. Typical band diagrams, calculated as transmittance colour maps, of each structure assuming a $\boldsymbol{x}$ (d-f) and $\boldsymbol{y}$ (g-i) polarized incident source. $k = |\boldsymbol{k}|$ is the magnitude of the parallel component (along the XY plane) of the wavevector and G_{11} is the magnitude of the corresponding lattice vector. The indexes $_h$ and $_P$ denote holes and single pillars, respectively. The black dots indicate the BICs for each band while the white arrow points towards the mode used for the experiments in the main text. The diameter (thickness) of the nano-holes W_h (t_h), single pillars W_P (t_P) and dimer pillars W (t) are, respectively, $W_h = 0.23\mathrm{a}_h$ ($t_h = 0.36\mathrm{a}_h$), $W_P = 0.91\mathrm{a}_P$ ($t_p = 0.28\mathrm{a}_P$) and $W = 0.37\mathrm{a}_y$ ($t = 0.21\mathrm{a}_y$). For the dimer pillar, $\mathrm{a}_x = 0.66\mathrm{a}_y$ and $g_c = 0.45\mathrm{a}_y$. For simplicity's sake, the simulations assumed a constant index for the aSi ($n = 3.5$), water as the cover material ($n = 1.33$) and a glass substrate ($n = 1.45$).

4. Resonance field profile dependence on g_c for dimer pillar structures.

The ratio between the electric field energy confined in the region of interest (RoI), highlighted in Fig. S4a, around the pillars U_E^{RoI} to the total electric field energy of the resonance can be calculated as:

$$U_E^{RoI} = \frac{\int_{RoI} \varepsilon(\boldsymbol{r})|\boldsymbol{E}(\boldsymbol{r})|^2}{\int_V \varepsilon(\boldsymbol{r})|\boldsymbol{E}(\boldsymbol{r})|^2} \tag{S14}$$

where $\boldsymbol{E}(\boldsymbol{r})$ is the electric field distribution, $\varepsilon(\boldsymbol{r})$ is the permittivity distribution and V is the unit cell volume, limited in the transversal region by the simulation boundaries. The dependence of this ratio on the distance g_c of the dimer pillar structure is shown in Fig. S4b (black solid line). As evident, changing g_c has a minimal impact on the energy field distribution of the modes supported by the dimer pillar, and therefore its sensing capabilities, especially for the high-Q

modes when g_c is close to $0.5a_y$. Meanwhile, g_c strongly affects the Q-factor of the modes, as shown in the blue dotted line of Fig. S4b. Examples of electric field distributions and transmittance spectra (ignoring absorption losses) for three different g_c distances (highlighted as 1, 2 and 3 in Fig. S4b) are shown in Fig. S4c-e. As evident by the similar field profiles in Fig. S4c-e, the dimer pillar allows for the fine control of the Q-factor without significantly impacting the field profile. Another interesting consequence of the stable field profile is the minimal change in resonance wavelength with the change of g_c (see the resonance wavelengths Fig. S4c-e), which indicates that the field overlap with the RoI - which determines the sensitivity - does not change appreciably.

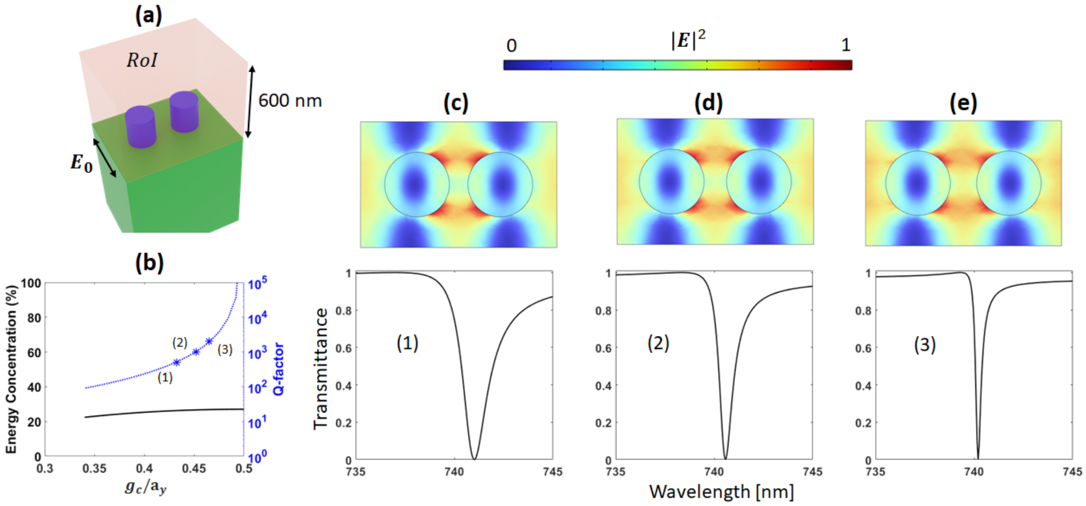

Figure S4: (a) Schematic of the dimer pillar unit cell and the Region of Interest (RoI) assumed for the energy concentration calculations. The RoI consists of a 600 nm thick layer above the structure. (b) The influence of g_c on the energy concentration inside the RoI (black solid line) and the Q-factor (blue dotted line). (c-e) Examples of electric field distributions and transmittance spectra (ignoring absorption losses) for three different g_c values highlighted as 1, 2 and 3 in (b), respectively.

5. Resonance shift of bulk sensitivity measurement

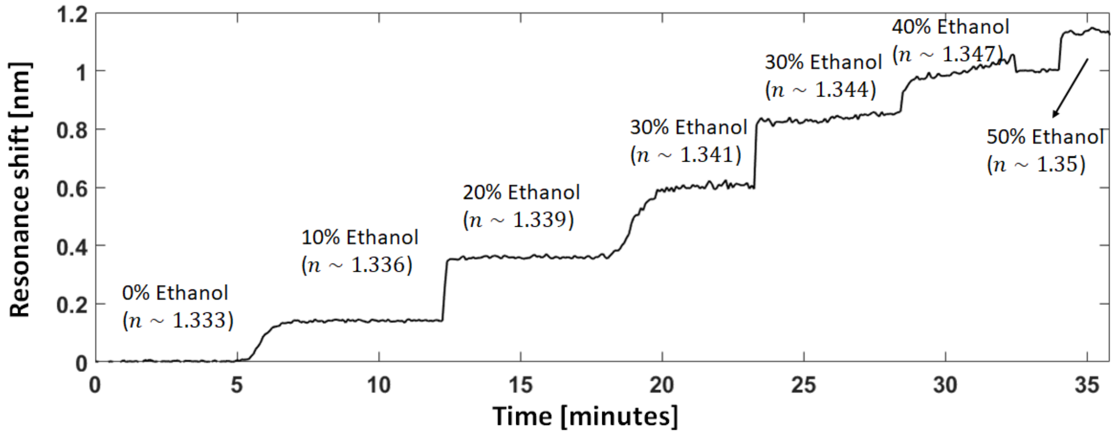

Figure S5: Resonance shift of bulk sensitivity measurement using the grating of Fig. 1 of the main paper.

6. Derivation of equation 2 of the main paper.

The Limit of Detection (LoD) of photonic resonances is given by [6]:

$$LOD = \frac{\lambda_0}{SQ_R A}\sqrt{3\sigma} \tag{S15}$$

where λ_0 is the resonance wavelength, S is the resonance sensitivity, given in nm/RIU, Q_R is the lossless cavity Q-factor (typically obtained via simulations), A is the amplitude of the resonance and σ is the standard deviation of the amplitude measurements (representing the noise of the experimental setup). The relation between the measured (Q) and resonating (Q_R) Q-factor is given by:

$$Q^{-1} = Q_R^{-1} + Q_{NR}^{-1} \tag{S16}$$

where Q_{NR} is the non-radiative Q-factor describing the losses. The relation between A, Q_R and Q_{NR} is given by:

$$A = \left(\frac{Q_R^{-1}}{Q_R^{-1}+Q_{NR}^{-1}}\right)^2 \tag{S17}$$

which can be rearranged as

$$1 + \frac{Q_{NR}^{-1}}{Q_R^{-1}} = \frac{1}{\sqrt{A}} \tag{S18}$$

From equations S18 and S16, one finds that

$$Q_R = \frac{Q}{\sqrt{A}} \tag{S19}$$

Inserting equation S19 into S15, the LoD can be written as:

$$LOD = \frac{\lambda_0}{SQ\sqrt{A}}\sqrt{3\sigma} \tag{S20}$$

Therefore, to improve the LOD, the denominator in equation S20 should be maximized, which we use the define our Figure of Merit (FOM). That is,

$$FOM \sim SQ\sqrt{A} \tag{S21}$$

which is equation 2 of the main text.

7. Extraction of the resonance Q-factor and amplitude.

To obtain the Q-factor (Q) and amplitude A of the measured resonances, the transmittance curves $T(\lambda)$ are fitted to a Fano function [7]:

$$T(\lambda) = a\frac{[q\varrho+(\lambda-\lambda_0)]^2}{\varrho^2+(\lambda-\lambda_0)^2} + e \tag{S22}$$

where a is the resonance amplitude ($A = a$), q is the Fano parameter [4,8], ϱ is the maximum half-width (such that full width at half maximum of the resonance is $\Delta_{FWHM} = 2\varrho$) , λ_0 is the peak wavelength and e is an offset value. From these parameters, the Q-factor can be obtained as:

$$Q = \frac{\lambda_0}{2\varrho} \tag{S23}$$

8. The orthogonal modes supported by the dimer pillar.

The photonic crystal supports two orthogonal sets of modes that can be assessed independently depending on the polarization of the incoming field. We label these modes following the slab waveguide nomenclature, that is, modes with field distributions primarily dominated by electric or magnetic field components orthogonal to their coupling directions are named quasi-transverse electric (TE-like) or quasi-transverse magnetic (TM-like) modes, respectively. In our design, both TE-like and TM-like are coupled along the **y** direction and can be excited with **x** (TE-like) or **y** (TM-like) polarized light (see Fig. S6). The electric field distributions for the TE-like and TM-like modes are shown in Fig. S6a and Fig. S6b, respectively. Their overlap with the water - which is related to the mode's sensitivity (S) - are similar for both TE-like and TM-like modes (see Fig. S6c, where it reaches 0.28 and 0.35 for the TE-like and TM-like modes, respectively). Note, however, that these two modes have very different field distributions: the TE-like mode is concentrated in the gaps between the pillars (Fig. S6a), while the TM-like mode is concentrated on top of the pillars (Fig. S6b).

The TM transmittance spectra measured and their corresponding product $Q\sqrt{A}$ of the dimer pillar structure of Fig. 2 of the main paper are shown in Fig. S6d-f for three different gap distances: 190, 200 and 210 nm. The highest product $Q\sqrt{A}$ operating with the qTM mode is obtained when Q $\sqrt{A}$ ~ 753 (Fig. S6e) for $g_c = 200\ nm$, a Q-factor of 1450 and A of 0.27. A further increase in Q-factor to 1600 (1.1x higher) by adjusting g_c to 210 nm (Fig. S6f) is accompanied by a reduction in the signal amplitude A to 0.19 (1.42x smaller), reducing the product to $Q\sqrt{A}$ ~ 697.

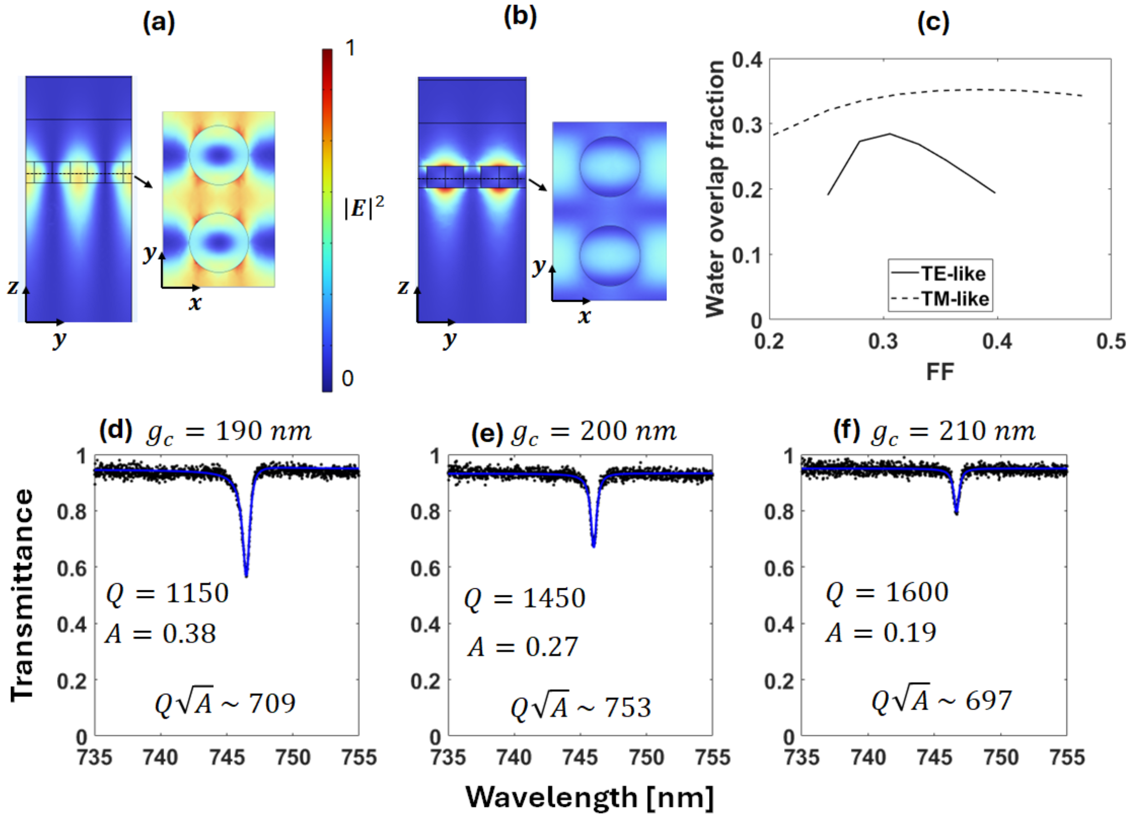

Figure S6: Electric field profiles of TE-like (a) and TM-like (b) modes. The $\boldsymbol{x}$, $\boldsymbol{y}$, and $\boldsymbol{z}$ directions are as defined in Fig. S1a. (c) Calculated water overlap field energy fraction for TE-like (solid line) and TM-like (dashed line) modes for different FF values. (d-f) Transmittance spectra measurements of TM-like modes of the structure used in Fig. 2 of the main paper for three different g_c values: 190, 200, 210 nm, respectively. The black dots represent the measurement data and the blue solid line is the Fano fitted curve used for extraction of the Q-factor and the amplitude A, which are displayed as inset values for each transmittance graph along their $Q\sqrt{A}$ product.

9. The bowtie chirped configuration.

The mode supported by the dimer pillar structure illustrated in Fig. 1c of the main text can be excited by a perpendicularly incident **x**-polarized light (see Fig. S7a) and its resonance wavelength (λ_r) is proportional to the period along the **y** direction (a_y), as given by:

$$\lambda_r = n_{eff} a_y(x) \qquad \text{(S24)}$$

where n_{eff} is the mode effective index. The grating period $a_y(x)$ is linearly chirped along the horizontal direction (**x**) but held constant along the vertical (**y**) direction. According to equation S24, the chirped array translates spectral information into spatial information ($\lambda_r \rightarrow$ x), which can be easily read-out using a digital camera. That is, at monochromatic illumination, the chirped array resonates only around the horizontal position that satisfies equation S24, which then can be seen as a bright vertical bar (highlighted in red in Fig. S7b and S7c). When the analyte binds to the grating surface, the mode effective index changes, which shifts the resonance to another period (different position in the chirp) since the wavelength is fixed. This displacement can then be recorded by a CMOS camera and post-processed to track its position. In our sensor (see Fig. 1 of the main text), the chirped grating is obtained by linearly tapering the period a_y from 496 nm to 504 nm over 500 μm along the **x** direction. These parameters result in resonances in the near visible range (~750 nm), where aSi has a low absorption coefficient [7]. A mirror copy of the chirped grating is placed next to the first one, resulting in a bowtie configuration, as illustrated in Fig. S7b. The readout setup consists of a telescope to demagnify and direct the beam towards the substrate with the grating on its surface, as illustrated in Fig. S7d (lens L1, with focus f_{L1}= 150 mm, and objective with focus $f_{obj} = 45\ mm$), and an inverted microscope (same objective f_{obj}, with another lens L2, with focus f_{L2}= 180 mm, and a beamsplitter) to image the sample into a digital camera, as illustrated in Fig. S7e. More information about the components can be found in the methods section of the main text.

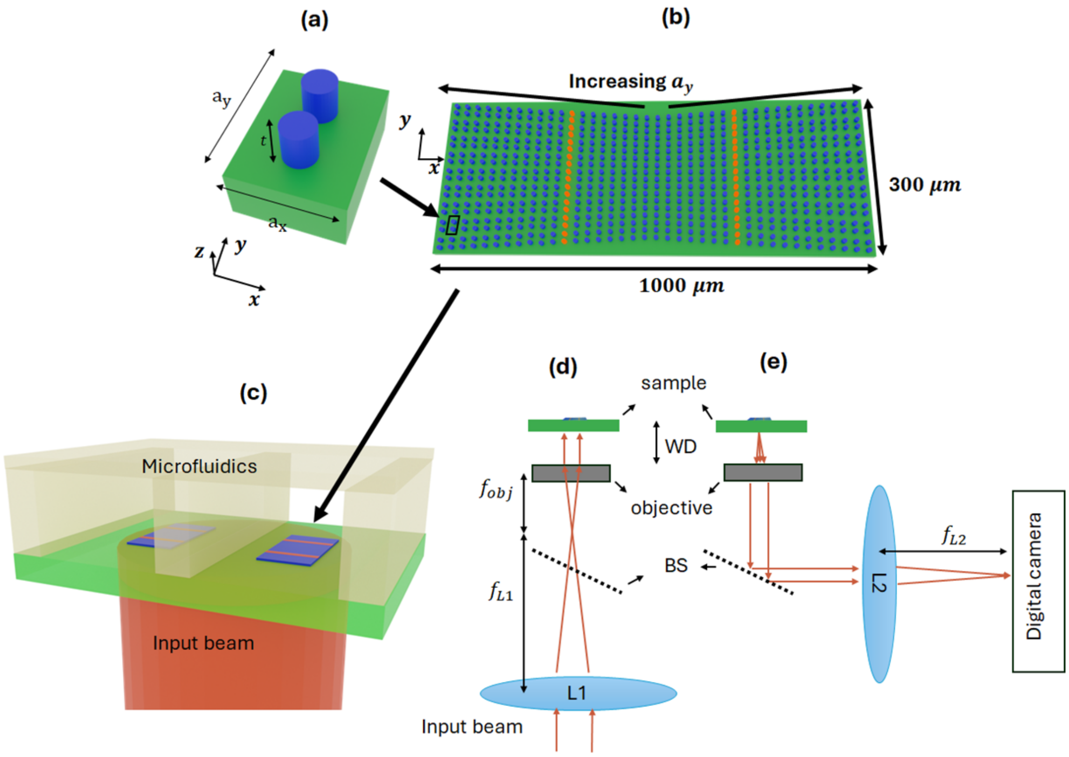

Figure S7: (a) Schematic of the unit cell of the dimer pillar array (thickness t and diameter W) with periods a_x and a_y in the corresponding $\boldsymbol{x}$ and $\boldsymbol{y}$ directions. (b) The bowtie chirped configuration used for biosensing. First, the chirped grating is obtained by tapering a_y along $\boldsymbol{x}$ between 496 nm and 504 nm over a distance of 500 μm; then, another mirrored chirped grating is added next to the first one, thus obtaining a bowtie configuration. Along the $\boldsymbol{y}$ direction, the unit cell with fixed a_y is repeated over a distance of 300 μm. When illuminated by normally incident monochromatic light (wavelength $\lambda_0 = 750\ nm$), the resonance manifests itself as a bright bar (in red) spatially located in the region where the ratio λ_0/a_y matches the mode's effective index (n_{eff}). (c) Microfluidic channels are added to deliver the chemical and biological reagents for the signal reading to different gratings on top of the glass substrate. The monochromatic source is perpendicularly incident through the glass substrate. The setup consists of an illumination path (d) and an imaging path (e) coupled via a beam splitter. The telescope in (d), which consists of a lens L1 (with focus f_{L1}) and an objective with focus f_{obj}, demagnifies the collimated beam and illuminates the sample. Reflected light by the grating is collected by the same objective and redirected by the beam splitter to a tube lens L2 (e), forming an image on a digital camera, effectively acting as an inverted microscope.

The spatial resonance shift is then measured as the difference in the relative distances between the two bars, which are tracked by fitting Lorentzian functions to each horizontal pixel line [9]. Such relative distances are highlighted as red arrows in Fig. 3d-f of the main paper. It is then possible to estimate the resonance spectral shift $\Delta\lambda_r$ from equation S24, such that:

$$\Delta\lambda_r = 0.5 n_{eff}(d_0 - d) r_{chirp} \qquad \text{(S25)}$$

where r_{chirp} is the chirp rate, defined as the ratio of grating period a_y variation along the horizontal x axis (four our case, $r_{chirp} =$ 0.016 nm/um), d_0 is the reference distance (measured before adding the antigen and AuNPs), d is the signal distance (due to the presence of the antigens or the AuNPs). The variation of n_{eff} is much smaller than the difference of the relative distances and it can be treated as a constant. For the resonances highlighted in Fig. 3d-f of the main paper, which are repeated here in Fig. S8a-c, the mean pixel column average intensity value is plotted against its horizontal position as black dots in Fig. S8d-f. Fano fittings were done for the two resonances on each side of the bowtie grating separately, and are shown as red curves n Fig. S8d-f. The $\sqrt{A}$ and Δ_{FWHM} (full width at half maximum of the resonance) values extracted from the fitted curves are displayed as inset values for each curve (the average value between both resonances is shown). Note that the $\sqrt{A}$ due to the AuNPs drops by a factor around 0.80 (from 6.4 – Fig. S8d – to 5.1 – Fig. S8f). Meanwhile, a 0.49x drop in Q-factor can be directly estimated by the broadening of the resonance given by the Δ_{FWHM} (from 66 – Fig. S8d – to 135 – Fig. S8f).

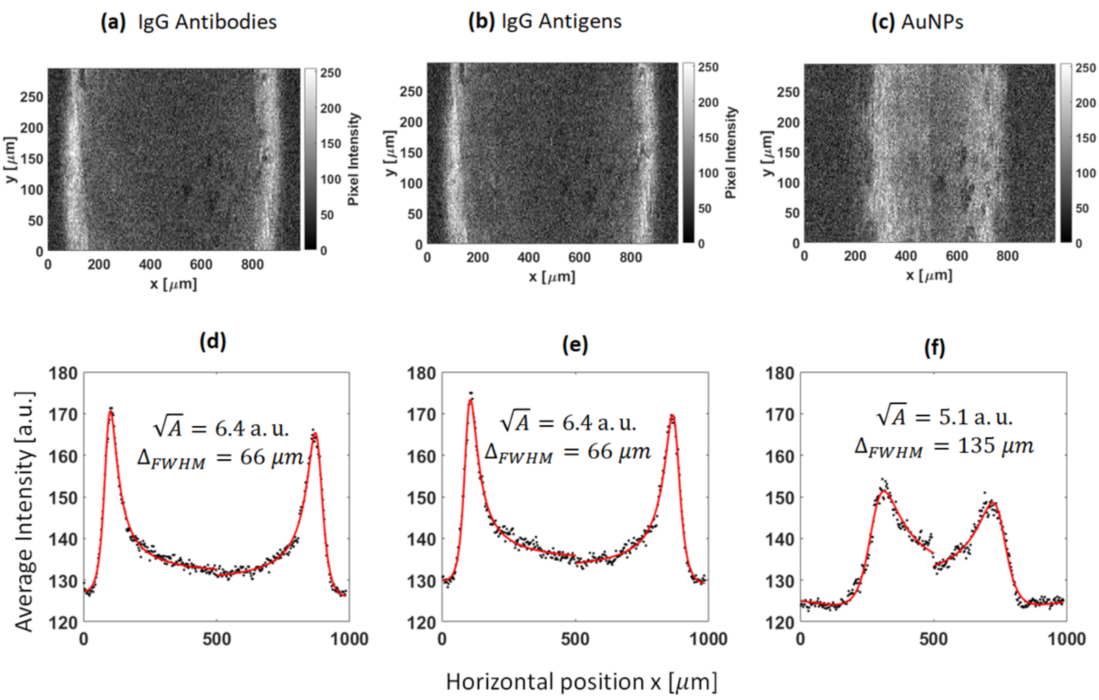

Figure S8: (a-c) Representative images of the sensor (raw camera data) at three different measuring stages of the experiment of Fig. 3 of the main text: after the addition of the surface IgG antibodies (a), the IgG antigen (b) and functionalized AuNPs (c). For the three cases, the average column pixel intensity value is plotted in (d-f), respectively, as black dotted curves while the Fano fitted curve used for the extracting the $\sqrt{A}$ and Δ_{FWHM} values are shown as the red solid line. The **x** and **y** directions are as defined in Fig. S7.

10. Sensor image and full-time scale experiments of Figure 4

Representative images of the sensor captured by the camera during the assay of the experiments of Fig. 4 of the main text are shown in Fig. S9a. We note that, since the peptide concentrations are in the pg/ml range, the binding of the functionalised AuNPs does not noticeably impact the resonance amplitude and Q-factor, contrary to the IgG experiments (where the concentration was 50 μg/ml), which clearly indicates the amplification capability of the nanoparticles. The full-time scale experiment of Fig. 4b of the main text is shown in Fig. S9b, where the solution of antibody-functionalized AuNPs was exhausted before all surface binding sites had been saturated, causing the observed resonance shift to terminate around 45 mins.

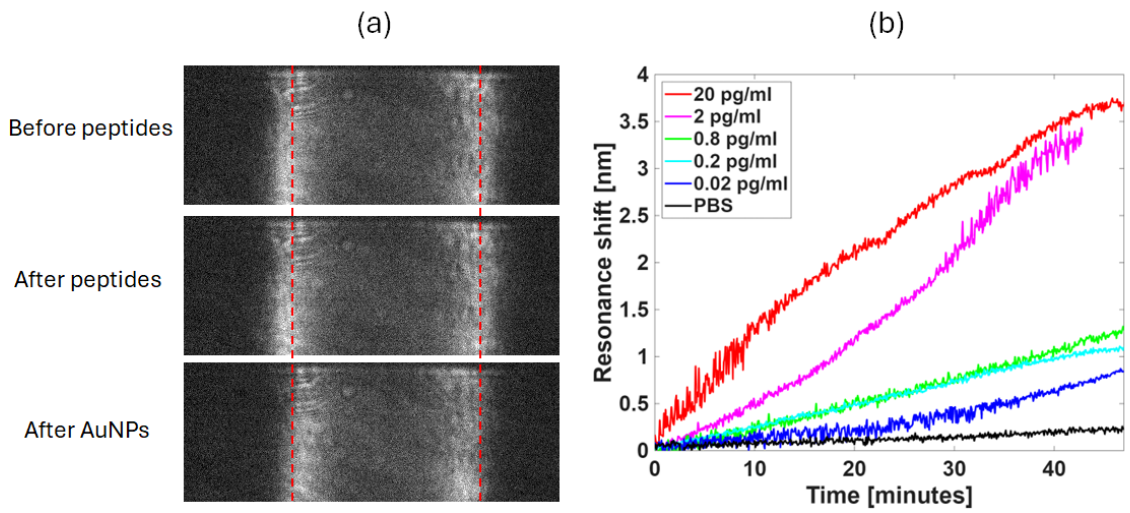

Figure S9: (a) Sensor's images captured by the camera at different assay stages: before and after the introduction of the peptides, followed by the introduction of the gold nanoparticles. The red dashed line is used as a guide to the eye. (b) Extended time trace data from the experiments of Fig. 4b in the main text. The traces represent the resonance shift upon the addition of antibody functionalised AuNPs for the following concentrations of Ab42 diluted in PBS: 20.0 (red), 2.0 (magenta), 0.8 (green), 0.2 (cyan) and 0.02 (blue) pg/ml. The measurement terminates around 45 minutes due to exhaustion of the antibody functionalised AuNP solution.

References:

1 Arruda, Guilherme S., et al. "Fourier Control of Air Modes in High-Q Metasurfaces." Advanced Optical Materials 12.3 (2024): 2301563. https://doi.org/10.1002/adom.202301563

2 Arruda, Guilherme S., et al. "Perturbation approach to improve the angular tolerance of high-Q resonances in metasurfaces." Optics Letters 47.23 (2022): 6133-6136. https://doi.org/10.1364/OL.475601

3 Whittaker, D. M., and I. S. Culshaw. "Scattering-matrix treatment of patterned multilayer photonic structures." Physical Review B 60.4 (1999): 2610. https://doi.org/10.1103/PhysRevB.60.2610

4 Fan, Shanhui, and John D. Joannopoulos. "Analysis of guided resonances in photonic crystal slabs." *Physical Review B* 65.23 (2002): 235112. https://doi.org/10.1103/PhysRevB.65.235112

5 Arruda, Guilherme S., et al. "Reducing the surface area of black silicon by optically equivalent structures." IEEE Journal of Photovoltaics 10.1 (2019): 41-45. https://doi.org/10.1109/JPHOTOV.2019.2945912

6 Conteduca, Donato, et al. "Beyond Q: the importance of the resonance amplitude for photonic sensors." ACS photonics 9.5 (2022): 1757-1763. https://doi.org/10.1021/acsphotonics.2c00188

7 Barth, Isabel, et al. "Phase noise matching in resonant metasurfaces for intrinsic sensing stability." *Optica* 11.3 (2024): 354-361. https://doi.org/10.1364/OPTICA.510524

8 Iizawa, Masatomi, et al. "The quantum and classical Fano parameter q." Physica Scripta 96.5 (2021): 055401. https://doi.org/10.1088/1402-4896/abe580

9 Li, Kezheng, et al. "Noise Tolerant Photonic Bowtie Grating Environmental Sensor." ACS sensors 9.4 (2024): 1857-1865. https://doi.org/10.1021/acssensors.3c02419
